# Evolution of Excitation–Inhibition Ratio in Cortical Cultures Exposed to Hypoxia

**DOI:** 10.3389/fncel.2018.00183

**Published:** 2018-07-03

**Authors:** Joost le Feber, Anneloes Dummer, Gerco C. Hassink, Michel J. A. M. van Putten, Jeannette Hofmeijer

**Affiliations:** ^1^Clinical Neurophysiology, TechMed Centre, University of Twente, Enschede, Netherlands; ^2^Biomedical Signals and Systems, TechMed Centre, University of Twente, Enschede, Netherlands; ^3^Department of Clinical Neurophysiology, Medisch Spectrum Twente, Enschede, Netherlands; ^4^Department of Neurology, Rijnstate Hospital, Arnhem, Netherlands

**Keywords:** electrophysiology, excitability, immunocytochemistry, synapse density, vesicular transporter proteins

## Abstract

In the core of a brain infarct, neuronal death occurs within minutes after loss of perfusion. In the penumbra, a surrounding area with some residual perfusion, neurons initially remain structurally intact, but hypoxia-induced synaptic failure impedes neuronal activity. Penumbral activity may recover or further deteriorate, reflecting cell death. Mechanisms leading to either outcome remain ill-understood, but may involve changes in the excitation to inhibition (*E*/*I*) ratio. The *E*/*I* ratio is determined by structural (relative densities of excitatory and inhibitory synapses) and functional factors (synaptic strengths). Clinical studies demonstrated excitability alterations in regions surrounding the infarct core. These may be related to structural *E*/*I* changes, but the effects of hypoxia /ischemia on structural connectivity have not yet been investigated, and the role of structural connectivity changes in excitability alterations remains unclear. We investigated the evolution of the structural *E*/*I* ratio and associated network excitability in cortical cultures exposed to severe hypoxia of varying duration. 6–12 h of hypoxia reduced the total synaptic density. In particular, the inhibitory synaptic density dropped significantly, resulting in an elevated *E*/*I* ratio. Initially, this does not lead to increased excitability due to hypoxia-induced synaptic failure. Increased excitability becomes apparent upon reoxygenation after 6 or 12 h, but not after 24 h. After 24 h of hypoxia, structural patterns of vesicular glutamate stainings change. This possibly reflects disassembly of excitatory synapses, and may account for the irreversible reduction of activity and stimulus responses seen after 24 h.

## Introduction

In the core of a brain infarct, loss of neuronal functioning is followed by irreversible neuronal damage and cell death within minutes. In the penumbra, an area surrounding the core with some remaining perfusion, neuronal activity is severely reduced, with no apparent structural damage (Symon et al., 1977; Bandera et al., 2006), and loss of neuronal activity is initially reversible. The penumbra may further deteriorate or recover during the first days after stroke, and therefore makes a promising target for therapeutic intervention to promote recovery. However, the mechanisms underlying development in either direction remain ill-understood.

The restricted availability of oxygen and glucose in the penumbra significantly limits the mitochondrial production of adenosine tri phosphate (ATP), the major energy source in the brain (Attwell and Laughlin, 2001). One of the early consequences of ATP depletion in the ischemic penumbra is synaptic failure (Hofmeijer and van Putten, 2012). However, synapses initially remain structurally intact, and if blood flow is restored in time, synaptic transmission failure is reversible. Failure occurs, at least in part, at the presynaptic side (Krnjević et al., 1991; Khazipov et al., 1995; Bolay et al., 2002) and has been associated with reduced endo- and exo-cytosis (Pathak et al., 2015; Fedorovich et al., 2017), decreased phosphorylation of presynaptic proteins (Bolay et al., 2002), and adenosine-mediated mechanisms (Khazipov et al., 1995; Sun et al., 2002). Impeded synaptic function generally leads to reduced neuronal activity in the penumbra (Symon et al., 1977; Hofmeijer and van Putten, 2012). While failure of excitatory synapses alone is sufficient for this reduction in neuronal activity, experimental evidence shows that hypoxia also depresses inhibitory synapses (Krnjević et al., 1991). It remains unclear if and how selectivity of synaptic vulnerability to hypoxia affects the excitation–inhibition ratio (*E*/*I* ratio) and excitability of neuronal networks.

In brain regions adjacent to stoke damage, most likely including the penumbra (Ramos-Cabrer et al., 2011), changes have been reported in the *E*/*I* ratio, which may have beneficial as well as adverse effects. Increased *E*/*I* ratios, achieved by pharmacological reduction of GABAergic signaling, may facilitate plasticity and thus be beneficial for motor recovery after stroke (Clarkson et al., 2010; Alia et al., 2016). Induction of central ischemic lesions in rats resulted in increased excitability in cortical areas (Luhmann et al., 1993; Mittmann et al., 1994; Schiene et al., 1996). An increased *E*/*I* ratio has been suggested to facilitate subthreshold inputs early after stroke, and thus to assist the remapping of function from damaged areas to peri-infarct surviving tissue (Murphy and Corbett, 2009). Clarkson et al. (2010) showed that after a stroke in mice, tonic neuronal inhibition was increased in the peri-infarct zone.

Conversely, several clinical studies have shown that disinhibition following stroke (Liepert et al., 2000; Manganotti et al., 2002; Swayne et al., 2008) may result in cortical hyper excitability, with an increased susceptibility to seizures (Burn et al., 1997; Bladin et al., 2000; Silverman et al., 2002; Naess et al., 2004). Thus, changes in *E*/*I* ratio, if beneficial at all, may carry behavioral costs (Jaenisch et al., 2016) and should be of transient character as later normalization of cortical excitability has been associated with good recovery of stroke patients (Manganotti et al., 2002; Swayne et al., 2008).

A recent study using dissociated cortical cultures suggested that excitatory neurons survive longer than inhibitory ones under hypoxic conditions (le Feber et al., 2016). This might in principle account for the observed disinhibition after stroke, but selective cell death would impede the normalization of excitation that is observed during later stages. Higher loss of transmission efficacy in inhibitory synapses during hypoxia seems a more plausible mechanism to account for a transient increase of the *E*/*I* ratio, but studies that aimed to verify this hypothesis yielded diverging results. Recordings in cortical slices exposed to transient oxygen deprivation showed a decrease in stimulus-evoked inhibitory post-synaptic potentials, suggesting failure of inhibitory synaptic transmission (Luhmann et al., 1993). However, a later study showed that excitatory synapses to hippocampal interneurons are especially vulnerable to hypoxia (Khazipov et al., 1995). Such selective ischemic vulnerability of glutamatergic synapses to inhibitory, GABAergic interneurons leads to elimination of inhibitory cortical input (Krnjević et al., 1991; Zhu and Krnjević, 1994), and would also be consistent with disinhibition.

In addition to changes in synaptic efficacy, hypoxia/ischemia induced low activity may also affect structural connectivity, that is, the density of excitatory and inhibitory synapses, via multiple pathways. Reduced synaptic activity may induce synapse elimination (Nguyen and Lichtman, 1996; Goda and Davis, 2003; Kano and Hashimoto, 2009). Alternatively, in response to low network activity, homeostatic mechanisms have been shown to upregulate the growth of axons (Schmitz et al., 2009) and dendrites (Wong and Ghosh, 2002), and the formation of spines and boutons (Florence et al., 1998). Both mechanisms may lead to alterations of the *E*/*I* ratio, and may thus underlie excitability changes. However, the effects of hypoxia/ischemia on structural connectivity have not yet been investigated, and the role of structural connectivity changes in excitability alterations remains unclear.

In the current study, we investigated the temporal development of the structural *E*/*I* ratio and excitability in cortical cultures during and after severe hypoxia (10% of normoxia) of varying duration. To quantify the relative densities of excitatory and inhibitory synapses, we applied immunocytochemical staining of vesicular glutamate (vGLUT) and GABA transporters (vGAT). This was complemented by electrophysiological recordings to assess the temporal evolution of network excitability under these conditions.

## Materials and Methods

### Cell Culture

Cortical cells were obtained from newborn Wistar rats (Janvier Labs, France). The cells were mechanically and chemically (trypsin) dissociated as described in (Stoyanova and Le Feber, 2014) and plated on polyethyleneimine (PEI) coated coverslips or microelectrode arrays (MEAs) containing 60 titanium nitride electrodes (Ø: 30; 200 μm pitch). The centers of coverslips and MEAs were coated with PEI. The plating concentration was 3–3.5 million cells/mL, which resulted in a fairly confluent monolayer of cells (∼2500 cells/mm^2^). Cultures were stored in an incubator under standard conditions of near 100% humidity, 5% CO_2_, and 36°C until experiments started after approximately 3 weeks. The culture medium was refreshed twice a week with (serum free) R12 culturing medium (Romijn et al., 1984). All procedures involving animals were conducted according to Dutch and European laws and guidelines, and approved by the Dutch Animal Use Committee (DEC).

### Induction of Hypoxia

Before experiments, cultures were moved from the incubator to a hypoxic chamber. Inside the chamber a humidified gas mixture of air and N_2_, completed with 5% CO_2_, was blown over the setup at an initial rate of 5 L/min, which was reduced to 2 L/min 20 min after the onset of a new setting. Mixtures of air and N_2_ could be delivered at any ratio and were computer controlled by Vögtlin red-y mass flow controllers. Normoxic conditions were realized by setting the flow controllers to 100% air, the hypoxic mixture contained 10% air and 90% N_2_. This reduced the partial oxygen pressure in the medium from ∼160 to ∼20 mmHg (le Feber et al., 2016). Cultures on coverslips were kept at 36°C using a HE002 heating pad ^1^, cultures on MEAs were placed in a 1060BC preamplifier with a TC02 temperature controller, set at 36°C (both Multi Channel Systems, Reutlingen, Germany). Cultures on coverslips were fixed directly after 6, 12, or 24 h of hypoxia, or 3 or 24 h after return to normoxia. Age matched control cultures were fixed at day *in vitro* (DIV) 18, 19, 20, 21, or 22, without being exposed to hypoxia. Cultures on MEAs were used to record spontaneous activity and responses to electrical stimulation before, during and after 6, 12, or 24 h exposure to hypoxia. All recordings from cultures on MEAs contained an initial normoxic period of 2–6 h (further referred to as baseline) before hypoxia, and were followed after return to normoxia for at least 6 h.

### Immunocytochemistry

After 10 min fixation with 4% paraformaldehyde in 0.1 M PBS, the cells were permeabilized for 30 min with 0.2% Triton X-100. After 30 min of NGS blocking buffer, the cells were incubated with the first antibodies (1:500 in PBS/NGS) overnight at 4°C. Subsequently, the cells were incubated with the appropriate secondary antibodies (1:500 in PBS) for 3 h at room temperature in the dark, followed by a DAPI (1:1000; Sigma-Aldrich, Cat. Nr. D9542-1MG) counterstaining for 10 min. Cells were mounted in Mowiol 4–88. After at least 3 h drying at room temperature in the dark, they were stored in the dark at 4°C. Primary antibodies used were: mouse anti vGLUT; Sigma-Aldrich (Cat. Nr. SAB5200262-100UG), rabbit anti vGAT; Sigma-Aldrich (Cat. Nr. V5764-200UL), rabbit anti synapsin1 (SYN1); Abcam (Cat Nr Ab64581), and mouse anti synaptophysin (SPH); Abcam (Cat Nr Ab11105). Secondary antibodies used were donkey anti mouse 555; Sigma–Aldrich (SAB4600060-250UL), goat anti mouse TR; Santa Cruz (sc-2983), and donkey anti rabbit FITC; Jackson ImmunoResearch (711-095-152).

Images were taken at 60× magnification using a Nikon Eclipse 50i Microscope and a Nikon DS-Fi1 digital camera. Some of these stainings were performed for validation purposes only: vGLUT and vGAT should both co-localize with SYN1 and SPH, but not with each other. Co-localization was quantified by Pearsons R (above threshold), using the coloc2 plugin in ImageJ. Synapses were counted by an in-house developed computer algorithm (see the Appendix for details).

### Electrophysiology

Results obtained from immuocytochemical stainings were combined with electrophysiological data that were previously obtained (le Feber et al., 2016). Data were recorded using custom made software (labview, National Instruments, Austin, TX, United States) driving a NI-PCI-6071E ADC board at a sample frequency of 16 kHz per channel. The array wide firing rate (AWFR) was determined as the total number of action potentials recorded at all electrodes during periods of spontaneous activity (50 min/h). Before each experiment two to three electrodes were selected for stimulation, based on their ability to trigger a clear network response in the 15–150 ms latency range (synaptically-mediated phase, see below). Cultures were electrically stimulated during the first 10 min of every hour using a STG1002 stimulus generator (biphasic current pulses, 200 μs per phase, 12–32 μA; inter pulse interval: 5–10 s). Stimulus responses typically consist of an early phase (0–15 ms latencies), dominated by action potentials that are directly induced by the stimulus current, and a late phase (15–150 ms latencies) that depends on synaptic transmission. This late phase is completely abolished after excitatory synaptic blockade (Fedorovich et al., 2017) and is therefore referred to as the synaptically-mediated phase of the response. For each stimulation period, we calculated the total number of action potentials (of all electrodes) in 5 ms bins, following stimulation of each electrode. We quantified the synaptically-mediated phase of the stimulus response by the area under the curve from 15 to 150 ms (*A*_Syn_). To enable comparison between experiments, for each culture, AWFR was normalized to its baseline value, and for each electrode *A*_Syn_ was normalized to its baseline value. To avoid unstable normalization, we set a threshold for *A*_syn_ (baseline) at six spikes.

### Statistical Analysis

Distributions of synaptic densities under different conditions were plotted to verify normality. Statistical significance of differences between groups was assessed by Student’s *t*-test. Possible differences between *E*/*I* ratios at different days under control conditions, as well as differences between control and hypoxic conditions were assessed by ANOVA. *p* < 0.05 was considered to indicate significant differences.

## Results

### Antibody Validation

**FIGURE 1:**
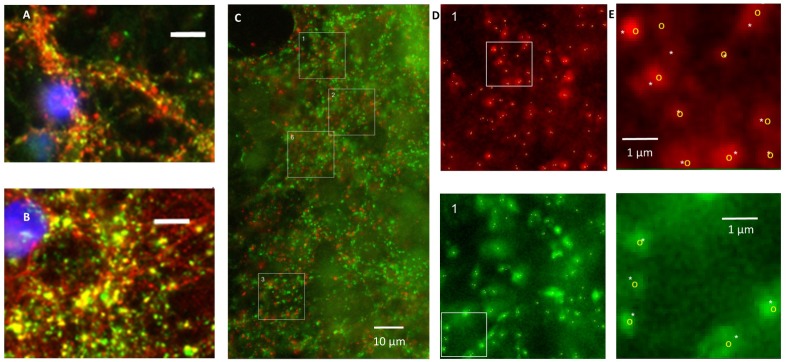
Validation of staining and counting of synapses. **(A)** Double staining of synaptophysin (red) and vGAT (green) showing that vGAT co-localizes with synaptophysin. **(B)** Double staining showing that vGLUT (red) co-localizes with synapsin 1 (green). **(A,B)** Blue: DAPI; scale bars: 10 μm. **(C)** Counting of excitatory and inhibitory synapses. Photos were obtained after 0, 6, 12, or 24 h of severe hypoxia (10% of normoxia). In all photos 15 μm × 15 μm areas were selected with a clearly scattered structure in the green channel and no artifacts. Selected areas are indicated as numbered white squares. In all selected areas the numbers of excitatory and inhibitory synapses were counted automatically. In addition, excitatory and inhibitory synapses in some areas were counted manually. **(D)** Magnifications of square 1, with counted synapses. Upper panels show vGLUT staining, lower panels show vGAT. **(E)** Magnification of the white bordered square in **(D)** to illustrate what was counted manually (indicated by white asterisks) and automatically (yellow circles).

Application of secondary antibodies without primary antibodies yielded no staining. vGLUT as well as vGAT staining yielded patterns of clearly separated small puncta ≤1 μm, often situated along neuronal cell bodies or processes (**Figures 1A–C**). In three cultures tested, vGAT co-localized with SPH (Pearson’s *R* = 0.62 ± 0.05, **Figure 1A**), and in three other cultures vGLUT co-localized with synapsin1 (**Figure 1B**). **Figure 1C** shows an example image of a culture stained for vGAT and vGLUT. We observed no co-localization of vGAT and vGLUT (Pearson’s *R* = 0.22 ± 0.03). vGLUT seemed to selectively stain the targeted proteins, vGAT also stained larger round areas (Ø ≈ 10–15 μm), similar to, but often more pronounced than in **Figure 1C**, top right. This also occurred without DAPI staining, and was therefore not caused by leakage from the blue channel (not shown). In all images, six 15 μm× 15 μm areas were selected that were artifact free in the green channel (see section “Materials and Methods”). In these areas there was always a good signal in the red channel, with scattered structures along somata and processes, except after 24 h of exposure to hypoxia. White squares indicate areas that were selected n this representative example to count the number of vGLUT positive (red) and vGAT positive (green) puncta. To reduce the time needed to count all puncta in the very large number of areas to be analyzed (>2000), we designed an algorithm for automated counting, which is described and validated in the Appendix.

### Synaptic Density Decreases, Excitatory Fraction Increases During Hypoxia

**FIGURE 2:**
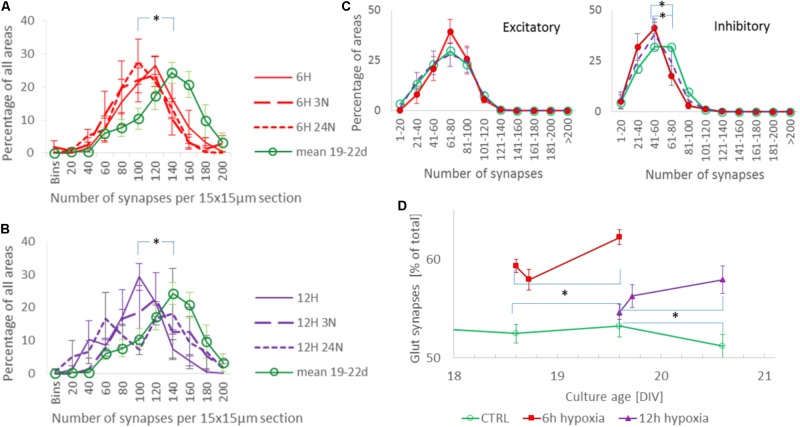
Evolution of synaptic density and the fraction of excitatory synapses during hypoxia. **(A,B)** Distribution of total synaptic density, obtained from 34 (normoxia) 12 (6H), 13 (6H3N), 10 (6H24N), 8 (12H), 6 (12H3N), and 6 (12H3N) cultures, in 4 (6 h hypoxia), 2 (12 h hypoxia), or 5 (normoxia) different staining sessions. Distributions were calculated from all counts of all cultures in each staining session, and averaged across staining sessions. Error bars show SEM and refer to differences between staining sessions. Mean synaptic density decreased following 6 **(A)** or 12 h **(B)** of hypoxia, as compared to normoxia (green line), and remained reduced for at least 24 h after re-oxygenation. **(C)** Density of excitatory (left) and inhibitory (right) synapses after 6 h (red solid line, filled circles) or 12 h (purple dashed line, no markers) of hypoxia, compared to these densities under control conditions (green solid line, open circles). Inhibitory synaptic density decreased significantly, while excitatory synaptic density did not. Cultures were fixed immediately after hypoxia, or 3 or 24 h after return to normoxia. Curves show averages per hypoxic condition, error bars represent SD and refer to differences between the moments of fixation. **(D)** The excitatory fraction of all synapses increased after 6 or 12 h of hypoxia, and remained higher than in control cultures for at least 24 h after re-oxygenation. ^∗^*p* < 0.001.

**FIGURE 3:**
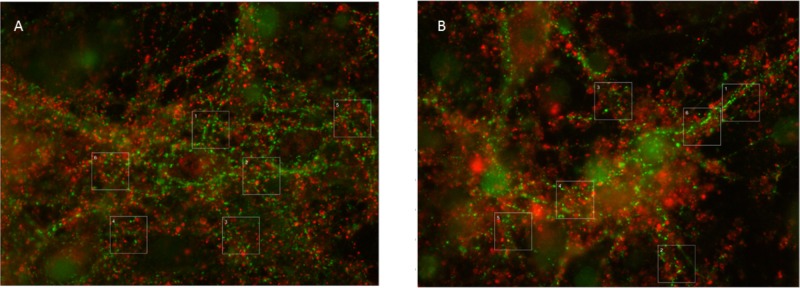
Typical examples of vGAT and vGLUT staining. **(A)** Under control conditions, vGLUT and VGLAT exhibited a scattered pattern, often along the edges of cell bodies or processes. In addition, vGAT staining occasionally showed larger green spots, which were excluded from analysis. **(B)** 24 h of hypoxia changed the scattered pattern of vGLUT positive puncta, to include large red areas. This new pattern was observed in all cultures exposed to 24 h of hypoxia, often, but not always, throughout the culture. It was never observed in cultures exposed to 6 or 12 h of hypoxia or in control cultures. vGAT positive puncta maintained their original structure, scattered along processes and somata.

The total synaptic density slightly but significantly decreased during six (**Figure 2A**; *t*-test: *p* < 0.001) or 12 h of hypoxia (**Figure 2B**; *p* < 0.001). This reflected a significant reduction of the inhibitory synapse density (*p* < 0.001), while excitatory synapse densities slightly increased (69.5 ± 19.7 vs. 67.2 ± 23.7) after 6 h of hypoxia (*p* < 0.01), or were unaffected after 12 h of hypoxia (*p* > 0.23). **Table 1** shows the number of cultures exposed to each condition, the number of photos taken and the number of analyzed areas. Densities remained significantly lower than that in control cultures not exposed to hypoxia for at least 24 h following return to normoxia (*p* < 0.001). **Figure 2C** provides an overview of the average density of excitatory (left) and inhibitory synapses after 6 or 12 h of hypoxia. In 786 analyzed areas, obtained from 32 cultures under normoxic conditions during 18–22 DIV, slightly more than 50% of all synapses were excitatory (52.5 ± 7.1%). There were no significant developmental changes during this experimentation period (ANOVA: *p* > 0.58; **Figure 2D**). The excitatory fraction of all synapses increased after 6 or 12 h of hypoxia, and remained higher than that in control cultures for at least 24 h following return to normoxia (ANOVA: *p* < 0.001; **Figure 2D**). After 24 h of hypoxia the excitatory fraction could only be determined in 10 of 89 photos taken from 16 cultures after two staining sessions. In the 60 analysis areas of these 10 photos, the excitatory fraction was still higher than in control cultures (63.5 ± 1.7%). In all other photos the excitatory synapses could no longer be counted properly; vGLUT could still be stained, but the stained puncta lost their original scattered structure along somata and processes, merging into much larger stained areas, see **Figure 3**. In lower density areas, counting was occasionally still possible, but no longer representative of the major part of the culture.

**Table 1:** Number of cultures used per condition.

Culture age	18 DIV	19 DIV	20 DIV	21 DIV	22 DIV
Cultures	5	8	9	7	3
Photos	17	32	39	31	13
Areas	102	186	234	186	78

	**Coverslips**				
**Hypoxia duration**	**Stained immediately**	**After 3 h of normoxia**	**After 24 h of normoxia**	**MEAs**	

6 h	12 Cultures	13 Cultures	10 Cultures	4 Cultures	
	54 Photos	52 Photos	44 Photos		
	324 Areas	324 Areas	264 Areas		
12 h	8 Cultures	6 Cultures	6 Cultures	5 Cultures	
	28 Photos	23 Photos	20 Photos		
	168 Areas	138 Areas	120 Areas		
24 h	7 Cultures	5 Cultures	4 Cultures	9 Cultures	
	35 Photos	34 Photos	20 Photos		
	30 Areas	6 Areas	24 Areas		

*Cultures on coverslips were exposed to normoxia (control, upper panel) or to hypoxia (10% of normoxia; lower panel) for 6, 12, or 24 h. Lower panel: cells were fixed immediately after hypoxia, or 3 or 24 h after reoxygenation, and stained for vGLUT and vGAT. Multiple photos were obtained from each culture without overlap. In each photo, up to six areas were selected for analysis, which showed the typical structure of stained puncta scattered along somata and processes, without artifacts. In cultures exposed to hypoxia for 24 h, less areas were selected, because the typical structure of stained puncta was largely lost. In parallel, cultures on MEAs were exposed to hypoxia of the same depth and duration, and activity and stimulus responses were recorded before, during and after hypoxia.*

### Spontaneous Activity and Responses to Electrical Stimulation Decrease and (Partially) Recover During Hypoxia

**FIGURE 4:**
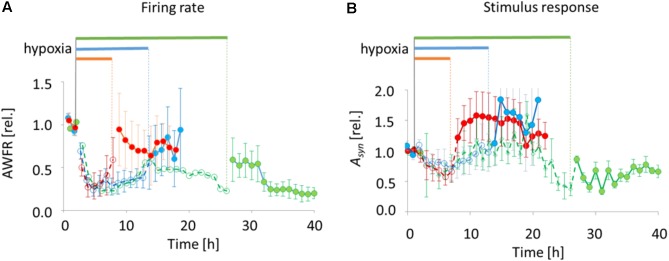
Total number of action potentials (AWFR) and synaptic phase of stimulus responses (the area under the curve of post-stimulus time histograms between 15 and 300 ms; *A*_syn_) before, during, and after exposure to hypoxia of varying duration. Curves were normalized to their mean value during baseline. **(A)** During the first hours of hypoxia, AWFR decreased to ∼25% of baseline activity, followed by partial recovery during hypoxia. Upon reoxygenation after 6 or 12 h, further recovery occurred. After 24 h, recovery was only partial. **(B)** The normalized synaptic phase of stimulus responses (*A*_syn_) also decreased during the first hours of hypoxia, followed by partial recovery. Upon return to normoxia after 6 or 12 h, *A*_syn_ further increased to values above baseline. This did not occur upon reoxygenation after 24 h. Horizontal bars above the curves and dashed lines with open circles indicate duration of hypoxia, solid lines with filled circles indicate pre- and post-hypoxia.

All 18 cultures included in the analysis were spontaneously active when experiments started, and generated firing patterns that included dispersed firing and synchronized network bursts. In 17 cultures recorded activity decreased rapidly following the onset of hypoxia (ANOVA, *p* < 0.001), to ∼25% of baseline. In one of the cultures exposed to 6 h of hypoxia, total activity during hypoxia was dominated by a single electrode with highly increased activity. The activity measured in this culture was considered to be an outlier, and excluded from further analysis. 6–12 h after the onset some activity tended to recover, toward ∼50% of baseline. Activity significantly recovered upon return to normoxia after 6 or 12 h (last 3 h before vs. first 3 h after reoxygenation; ANOVA *p* < 0.04), reaching 60–100% of baseline, but not after 24 h (reaching 20–50% of baseline; **Figure 4A**).

Stimulus responses were recorded before, during and after 6 h (9 electrodes stimulated in 4 cultures), 12 h (11 electrodes stimulated in 5 cultures), or 24 h of hypoxia (8 electrodes of 3 cultures stimulated). The synaptically-mediated phase of stimulus responses, as quantified by *A*_syn_, decreased during the first 5 h of hypoxia (ANOVA: *p* < 0.03) to ∼50% of baseline responses (**Figure 4B**). Thereafter, during hypoxia, stimulus responses recovered to values close to baseline responses. Upon return to normoxia after 6 or 12 h, stimulus responses became potentiated and remained potentiated for at least 7 h (baseline vs. first 7 h after reoxygenation; ANOVA: *p* < 0.05). After 24 h of hypoxia stimulus responses remained significantly below prehypoxia values (baseline vs. first 7 h after reoxygenation; ANOVA: *p* < 0.001).

## Discussion

In this study, we determined the effect on synaptic density and the excitatory/inhibitory synapse ratio in cortical neural cultures after exposure to hypoxia. We show a decrease of total synaptic density with an increased *E*/*I* ratio in cultured neuronal networks exposed to hypoxia up to 24 h. The dominant change after hypoxia was reduction in the inhibitory synaptic density, in agreement with recent results obtained in hippocampal cultures (Hwang et al., 2017). The increased *E*/*I* ratio suggests increased excitability, consistent with earlier findings *in vivo* (Liepert et al., 2000; Manganotti et al., 2002; Swayne et al., 2008) and *in vitro* (Luhmann et al., 1993; Mittmann et al., 1994; Schiene et al., 1996), that indicated increased excitability due to decreasing inhibition. The increased excitability only became apparent upon reoxygenation, with higher stimulus responses than during baseline. Presumably, the higher fraction of excitatory synapses did not result in higher excitability during hypoxia due to wide spread hypoxia-induced synaptic failure (Khazipov et al., 1995; Bolay et al., 2002; Hofmeijer et al., 2014; le Feber et al., 2017). If reoxygenation occurs in time, synaptic function recovers (le Feber et al., 2016, 2017), and increased excitability becomes apparent, given that the *E*/*I* ratio does not immediately re-adapt. **Figure 2D** shows that re-adaptation does not occur within 12 h after reoxygenation, possibly due to the fact that network activity does not increase above baseline. Increased activity may be required as a driving force to initiate re-adaptation.

Synaptic vulnerability may refer to the extent of acute synaptic depression during hypoxia, which probably differs between excitatory and inhibitory synapses (Khazipov et al., 1995), or to synapses’ susceptibility to elimination or pruning. Depression may lead to elimination through multiple pathways. Synapse elimination has been shown to be triggered by synapse inactivity (Nguyen and Lichtman, 1996; Goda and Davis, 2003; Kano and Hashimoto, 2009), suggesting that elimination may occur secondary to becoming obsolete after sustained synaptic failure. In addition, the formation and elimination of synapses is regulated by homeostatic processes that aim to maintain network activity within a certain range (Butz and van Ooyen, 2013). This mechanism may lead to a further reduction of inhibitory synapses following periods of low activity, and possibly to upregulation of the number of excitatory synapses.

### Possible Mechanisms Underlying Altered *E*/*I* Ratios

The simplest mechanism that might explain the increased *E*/*I* ratio, would assume higher susceptibility of inhibitory synapses to hypoxia-induced pruning, combined with large scale, reversible synaptic depression. However, earlier work in hippocampal slices suggested that certain excitatory synapses were more vulnerability to transient anoxia than inhibitory synapses (Khazipov et al., 1995). Therefore, it seems improbable that the activity of inhibitory synapses dropped further than that of excitatory synapses, and inactivity induced synapse pruning would probably affect excitatory synapses more than inhibitory ones.

Activity homeostasis seems a more plausible mechanism, as failure of excitatory synapses rapidly leads to decreasing activity (Hofmeijer et al., 2014; le Feber et al., 2016, 2017). It seems unlikely that the reduced activity resulted from increased inhibition because we saw decreased activity at all electrodes in almost all experiments. Approximately 20% of the neurons in dissociated cortical cultures are inhibitory, and increased inhibition should therefore become visible as increased activity at ∼20% of the active electrodes. One of the 18 experiments showed increased activity at a single electrode. This number is too low to represent increased activity in the inhibitory population, and the focally increased activity in this experiment was more likely to result from disinhibition (le Feber et al., 2016). Under normoxic conditions, homeostatic mechanisms have been shown to respond to periods of low activity by upregulation of excitatory synapses (Turrigiano et al., 1998) and downregulation of inhibitory ones (Kilman et al., 2002). At longer timescales, also structural changes may occur, as additional excitatory synapses are formed and inhibitory ones may be pruned (Butz and van Ooyen, 2013). Similar processes may occur during hypoxia: large scale reversible synaptic failure reduces total activity and consequently renders several (inhibitory as well as excitatory) synapses obsolete and thus prone to elimination. The increasing fraction of excitatory synapses might then reflect activity homeostatic mechanisms. A recent modeling study confirmed that slow recovery from large scale synaptic depression in combination with hypoxia-induced potentiation of excitatory synapses could reproduce a range of EEG patterns, as well as transitions between patterns that are commonly recorded from comatose patients after transient anoxia due to cardiac arrest (Ruijter et al., 2017).

### Glutamate Transporters Lose Their Scattered Structure Along Somata and Processes After 24 h of Hypoxia

Thus, elimination of excitatory synapses may have occurred at a slower rate due to compensatory upregulation of excitatory synaptic activity. This, however, seems to rescue excitatory synapses only for a limited period of time. vGLUT proteins, but not vGAT, lost their original scattered structure along the soma and processes of neurons after 24 h of severe hypoxia (**Figures 3A,B**). This was a robust structural change, observed in all cultures exposed to 24 h of hypoxia, often throughout the culture. We never saw similar structural changes in cultures exposed to shorter periods of hypoxia. The robustness of these findings, and the observation that vGAT staining continued to yield the same structural patterns, make it unlikely that the observed differences reflected a staining artifact, and support the conclusion that 24 h of hypoxia triggered a driving force on excitatory synapses that changed the structural pattern of vGLUT. We can only speculate on the underlying mechanism. One possible mechanism might be synapse disassembly. The exact nature of this process remains ill-known, most of the facts known about how synapses disassemble have been attributed to studies at the neuromuscular junction. Examination of fixed preparations at the fly neuromuscular junction suggests that retraction of the microtubule cytoskeleton may be one of the earliest events during synapse retraction (Eaton et al., 2002). If a similar mechanism applies to cortical synapses, this might explain the observed structural changes. In hippocampal cultures, higher fractions of synaptic proteins at extrasynaptic sites were recently demonstrated, associated with synapse disassembly (Leshchyns’ka et al., 2015).

### Limitations

Networks of dissociated cortical neurons are usually cultured under atmospheric “normoxic” conditions, at four to five times higher partial oxygen pressure than reported *in vivo* (Nair et al., 1987; Grote et al., 1996). It is uncertain how this relatively high pO_2_ under *in vitro* conditions relates to physiological oxygen pressure *in vivo*. Still, electrophysiological activity of all cultures responded immediately to decreasing pO_2_, indicating a clear effect of pO_2_ variations in the applied range, as discussed before (le Feber et al., 2016).

The random structure of networks grown from dissociated neurons may have affected vulnerability to ATP depletion. Particularly astrocyte support is important and may be different in 2D dissociated networks. Neurons are far more susceptible to ischemic damage than astrocytes, but astrocytes provide essential metabolic support to neurons during transient ischemia (Rossi et al., 2007; Takano et al., 2009). Experiments in mouse hippocampal slices showed that during hypoxia, astrocytes contribute to regulate excitatory synaptic transmission through the release of adenosine, which reduces presynaptic transmitter release (Martín et al., 2007). Conversely, astrocytes were able to restore neuronal activity under conditions of glucose deprivation due to lactate provided by the astrocytes (Rouach et al., 2008). In the current study, however, this ability seems less relevant, since only oxygen was limited, not glucose. Although structural differences may have affected results (e.g., 2D vs. 3D structures), astrocyte density in the cultures used (ratio astrocytes: neurons≈ 4:1 (le Feber et al., 2017) closely matched that reported in cortex *in vivo* (Azevedo et al., 2009).

In the ischemic penumbra *in vivo*, the availability of both glucose and oxygen are restricted, and additional processes, possibly related to the limited availability of glucose, may occur in parallel to the processes observed in the current study. Furthermore, neurons in the peri-infarct zone may also become deprived of input from the infarct core and other silenced areas. In addition to cortical excitatory and inhibitory circuits, afferent input has also been suggested to modulate cortical (Le Feber et al., 2014) and hippocampal excitability (Dyhrfjeld-Johnsen et al., 2010).

## Conclusion

We found that severe hypoxia reduced the total synaptic density. The inhibitory synaptic density decreased significantly more than the excitatory synaptic density, which yielded an increased *E*/*I* ratio, and, in principle, a more excitable network. Increased excitability was initially masked by large scale (reversible) synaptic failure, and became apparent only after return to normoxia. The increased *E*/*I* ratio probably reflects activity homeostatic mechanisms, triggered by hypoxia-induced low activity. If hypoxia lasts too long, permanent damage occurs, possibly due to disassembly of excitatory synapses.

## Author Contributions

JlF, JH, and MvP made a substantial contribution to the study concept and design. AD and GH did the acquisition of data. JlF did the analysis and interpretation of data and drafted the article. All authors revised it critically for important intellectual content and approved the version to be published.

## Conflict of Interest Statement

The authors declare that the research was conducted in the absence of any commercial or financial relationships that could be construed as a potential conflict of interest.

## Figures and Tables

**FIGURE 5:**
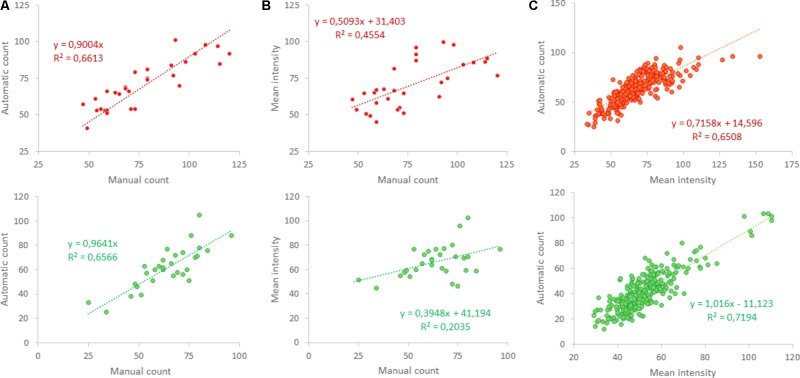
Validation of automated counting. **(A)** Shows that automated counting correlated well to the manually counted number in 30 photos obtained from five different cultures in the red (upper panel) as well as the green channel (lower panel); *R*^2^ > 0.65. **(B)** Mean intensity also correlated to the manually counted number, but with lower correlation coefficients [*R*^2^ = 0.46 (red) and *R*^2^ = 0.2 (green)], even using two-parameter regression. **(C)** Automated counts were compared to mean intensity in 288 areas in 48 photos obtained from 12 different coverslips and showed clear correlation (*R*^2^ > 0.65).

## Footnotes

1 Vet-tech.co.uk

## Appendix

### Automated Synapse Counting Algorithm

First, all intensities were scaled to cover the full range of intensities (0–255). Thresholds for detection of synapses were set at one third of the range (= 85) for red (vGLUT) and green (vGAT). It was usually possible to select six 250 × 250 pixel squares (15 μm × 15 μm) without clear artifacts from each image for counting. The only exceptions were images obtained after 24 h of hypoxia.

In each selected square the intensities of both colors were scaled to the full range again. Then, a 2D moving average filter (9 × 9 filtering kernel, all equal weights with sum 1) was applied to smoothen intensity variations around the centers of stained puncta. Local maxima were determined as regions of equal intensity that were completely surrounded by pixels with lower intensity. Finally, all selected squares were scanned from left to right and from the top–down to find pixels that were part of a local maximum. If the intensity of that pixel was above the threshold set for that color, it was counted as a synapse. The 9 pixels following to the right and to the bottom were discarded as local maxima to avoid double counting.

In 30 selected squares, obtained from five different cultures, excitatory and inhibitory synapses were also counted manually to validate the counting algorithm. Similarity between manual and automated counting was quantified by the squared correlation coefficient *R*^2^. **Figure 1D** shows the red and green channel of the first selected area in **Figure 1C**, as well as manual (indicated by white “^∗^”) and automated counts (yellow “o”). Manual and automated counting usually counted the same puncta (**Figure 1E**), and generally yielded very similar results. Automatic counting tended to slightly underestimate the number of positive puncta, particularly in the red channel (**Figure 5A**). As an alternative estimate of the density of positive puncta, the mean intensity of red and green were evaluated. However, this yielded much lower correlation coefficients (*R*^2^ = 0.46 and *R*^2^ = 0.20; **Figure 5B**) than between manual and automatic counting (*R*^2^ = 0.66 for red and green, **Figure 5A**). Still, the average intensity correlated well with the automatically counted number (**Figure 5C**).
